# Primary and Memory Response of Human Monocytes to Vaccines: Role of Nanoparticulate Antigens in Inducing Innate Memory

**DOI:** 10.3390/nano11040931

**Published:** 2021-04-06

**Authors:** Mayra M. Ferrari Barbosa, Alex Issamu Kanno, Leonardo Paiva Farias, Mariusz Madej, Gergö Sipos, Silverio Sbrana, Luigina Romani, Diana Boraschi, Luciana C. C. Leite, Paola Italiani

**Affiliations:** 1 Laboratório de Desenvolvimento de Vacinas, Instituto Butantan, São Paulo, SP 05503-900, Brazil; mayra_mara@msn.com (M.M.F.B.); alex.kanno@butantan.gov.br (A.I.K.); 2 Laboratório de Inflamação e Biomarcadores, Instituto Gonçalo Moniz, Fundação Oswaldo Cruz, Salvador, BA 40296-710, Brazil; leonardo.farias@fiocruz.br; 3 Istituto di Biochimica e Biologia Cellulare, Consiglio Nazionale delle Ricerche, 80131 Napoli, Italy; mariusz.madej@ocello.nl (M.M.); gergoosipos@hotmail.com (G.S.); 4 Istituto di Fisiologia Clinica, Consiglio Nazionale delle Ricerche, 54100 Massa, Italy; silverio.sbrana@ifc.cnr.it; 5 Dipartimento di Medicina e Chirurgia, University of Perugia, 06132 Perugia, Italy; luigina.romani@unipg.it; 6 Stazione Zoologica Anton Dohrn, 80121 Napoli, Italy

**Keywords:** innate immunity, innate memory, *Schistosoma mansoni*, monocytes, macrophages, vaccination

## Abstract

Innate immune cells such as monocytes and macrophages are activated in response to microbial and other challenges and mount an inflammatory defensive response. Exposed cells develop the so-called innate memory, which allows them to react differently to a subsequent challenge, aiming at better protection. In this study, using human primary monocytes in vitro, we have assessed the memory-inducing capacity of two antigenic molecules of *Schistosoma mansoni* in soluble form compared to the same molecules coupled to outer membrane vesicles of *Neisseria lactamica*. The results show that particulate challenges are much more efficient than soluble molecules in inducing innate memory, which is measured as the production of inflammatory and anti-inflammatory cytokines (TNFα, IL-6, IL-10). Controls run with LPS from *Klebsiella pneumoniae* compared to the whole bacteria show that while LPS alone has strong memory-inducing capacity, the entire bacteria are more efficient. These data suggest that microbial antigens that are unable to induce innate immune activation can nevertheless participate in innate activation and memory when in a particulate form, which is a notion that supports the use of nanoparticulate antigens in vaccination strategies for achieving adjuvant-like effects of innate activation as well as priming for improved reactivity to future challenges.

## 1. Introduction

In vaccine development, antigen-specific immune responses, and the development of long-term protective immunological memory are currently sought by exploiting technological platforms to construct vaccines that allow for appropriate antigen presentation. No vaccines are currently available for the parasite *Schistosoma mansoni*, which is the causative agent of schistosomiasis that is one of the most devastating parasitic diseases in terms of public health and socio-economic impact [1]. Among the most promising *S. mansoni* antigens that are currently considered for vaccine development are the two surface proteins SmCD59.2 and SmTSP-2. SmCD59.2 is a GPI-anchor tegument surface-exposed immunogenic protein [2] orthologue of human CD59 [3], whose function remains to be established [2,3,4]. At variance with human CD59, SmCD59.2 does not show any activity on complement [3]. Whether SmCD59.2 could interact with innate receptors, as it has been shown for the human orthologue [5], is currently unknown. SmTSP-2 is an immunogenic tetraspanin protein [2] essential in schistosomula development [4], with structural properties in cell membrane orgnization and in the parasite’s tegument [6]. Based on its similarity with human tetraspanins, it is hypothesized that SmTSP-2 could interact with integrins and MHC-II on human cell membranes [4]. Both molecules have been suggested as potential vaccine candidates, although the results to date indicate the need to increase their immunogenicity [7,8,9]. To this end, these proteins were expressed in recombinant form in fusion with the biotin-binding protein rhizavidin and coupled to biotin-labeled Outer Membrane Vesicles (OMV) of *Neisseria lactamica*, thereby generating antigen-decorated OMV that were more effective in inducing antigen-specific humoral and cellular immunity in mice compared to the soluble protein alone [10,11]. In fact, mice immunized with the antigen-decorated OMV could generate an antigen-specific IgG antibody response much more potent than that induced by the soluble antigen or by the mixture of soluble antigen with bare OMV, the antibody production being paralleled by antigen-specific activation of CD4^+^ and CD8^+^ T lymphocytes in the spleen [10,11].

In assessing the efficacy of vaccine candidates, it is also important to evaluate the effects on innate immunity, i.e., the host response that, although not directly responsible for antigen-specific recognition, reaction, and specific protective memory, has a central role in amplifying antigen-specific responses and in establishing effective long-term immunity. In this perspective, the role of vaccine adjuvants is that of activating innate immunity to improve vaccine efficacy. Similar to adaptive immunity, innate immunity can display memory, i.e., a variation in the secondary response to a challenge, which depends on the host being previously exposed to/primed with the same or other agents [12,13,14,15,16]. At variance with adaptive memory, which is antigen-specific, innate memory is largely non-specific in mammals, with exposure to a given stimulus (e.g., bacterial LPS) causing a secondary memory response that is the same to a variety of different agents [12,13,14,15,16]. Innate memory responses aim at shaping the innate/inflammatory response to secondary challenges in a way that is more protective and less damaging than the first reaction, as in the case of LPS tolerance that limits the extent of the local inflammatory reaction, which would cause significant damage to the affected tissue, while maintaining the production of chemokines and alarmins that initiate the defensive immune reaction [12,17,18,19,20,21,22]. Most interestingly, vaccination with several whole live attenuated vaccines (*B. pertussis*, BCG, poliovirus, smallpox, measles, measles–mumps–rubella) was shown to induce long-term resistance not only to the specific immunizing microorganism but also to different pathogens [23,24,25,26], suggesting that vaccine-induced non-specific innate memory can amplify vaccine-induced protection by extending it to non-related infections. It is notable that particulate agents are very efficient in inducing innate memory, to underline the fact that cells of the innate immune system, in particular mononuclear phagocytes such as monocytes and macrophages, can recognize size/shape in addition to molecular patterns [27].

In this study, we have used an in vitro system based on human primary monocytes to assess the capacity of *S. mansoni* antigens, either soluble or displayed on the OMV surface, to induce innate primary and memory responses, in order to examine the possible contribution of innate immunity in the overall efficacy of the anti-*S. mansoni* candidate vaccines. Our data show that the soluble Sm antigens do not induce significant innate activation of either monocytes or monocyte-derived macrophages in terms of production of the inflammatory cytokines TNFα and IL-6, and of the anti-inflammatory cytokine IL-10, while both bare and antigen-displaying OMV are effective although to different extents. Priming with soluble rSmTSP-2 or rSmCD59.2 did not induce memory to either the same or an unrelated challenge (LPS). Priming with bare or antigen-decorated OMV induced a significant tolerance in terms of TNFα production and a significant IL-10 production in response to LPS but no substantial changes in response to the homologous stimuli. As a control for assessing the role of size in inducing innate memory, the primary and memory TNFα response of human monocytes and macrophages to LPS from *Klebsiella pneumoniae* was compared to that to whole *K. pneumoniae* bacteria. Although LPS is a potent stimulus of innate responses, also in this case the memory response induced by whole bacteria was more pronounced than that induced by purified LPS.

## 2. Materials and Methods

### 2.1. Synthesis and Characterization of rRzv:SmCD59.2 and rRzv:SmTSP-2 OMV Complexes

The recombinant fusion proteins between rhizavidin from *Rhizobium etli* and *S. mansoni* CD59.2 and TSP-2 (rRzvSmCD59.2 and rRzvSmTSP-2) were produced in *E. coli*, purified, and characterized as previously described in detail [10,11]. The elimination of possible contaminating LPS was performed with the Triton X-114 wash method [28], yielding recombinant proteins with an LPS contamination <8.0 EU/mg, as evaluated by the LAL gel-clot assay (Lonza Group Ltd., Basel, Switzerland). OMV were obtained from *Neisseria lactamica* 799/98, purified and detoxified by treatment with sodium deoxycholate, and shown to reduce the LPS content by 95% [29]. LPS in membranes is considered 100x less toxic than free LPS [30]; thus, the LPS activity rather than amount was always measured to meet the quality control criteria for OMV vaccines that set the limit of LPS activity to <400 EU/μg. However, the 2-keto-3-deoxy-D-mannooctanoic acid (KDO) measurement (see below) performed on some OMV samples confirmed the presence of an amount of LPS that matched the measured activity, i.e., 1 EU = 0.1 nanogram. Biotinylated OMV were obtained as previously described [10]. Briefly, 25 mg OMV were incubated with 10 mg biotin in sodium phosphate buffer, 150 mM NaCl, 3% sucrose, and 0.1 M *N*-(3-dimetylaminopropyl)-*N*’-ethylcarbodiimide hydrochloride, and re-purified by gel filtration chromatography [10]. Recombinant proteins were coupled to biotinylated OMV by exploiting biotin–rhizavidin affinity binding, as previously described for the Multiple Antigen Presenting Strategy (MAPS) [31]. Avidin binding to biotin is the strongest non-covalent interaction known in nature, and it has been extensively developed and approved for many therapeutic applications, including cancer treatments [32,33]. Biotinylated OMV were incubated with rRzvSmCD59.2 or rRzvSmTSP-2 for 18 h at 4 °C at a 5:1 mass ratio and then purified by size exclusion chromatography on Sephacryl S-200 in endotoxin-free conditions [10]. The antigen to OMV protein mass ratio after conjugation was 1:10–1:20 [11].

The OMV-protein complexes, displaying SmCD59.2 (OMV:D) or displaying SmTSP-2 (OMV:T) and the unconjugated purified biotinylated OMV (OMV), were characterized by transmission electron microscopy (TEM) using a JEM-1230 microscope (JEOL Ltd., Tokyo, Japan), and for electrophoretic mobility, and for hydrodynamic size/polydispersion by dynamic light scattering (DLS) with a Zetasizer Nano ZS90 instrument (Malvern Panalytical Ltd., Malvern, UK) with a fixed scattering angle of 173° at 25 °C in triplicate [9]. Endotoxin/LPS contamination was assessed with the gel clot LAL assay (Lonza Group Ltd., Basel, Switzerland) and with the 2-keto-3-deoxy-D-mannooctanoic acid (KDO) assay.

### 2.2. Human Monocyte Isolation and Differentiation of Monocyte-Derived Macrophages

Blood was obtained from healthy donors upon informed consent and in agreement with the Declaration of Helsinki. The protocol was approved by the Regional Ethics Committee for Clinical Experimentation of the Tuscany Region (Ethics Committee Register n. 14,914 of 16 May 2019). Monocytes were isolated by CD14 positive selection with magnetic microbeads (Miltenyi Biotec, Bergisch Gladbach, Germany) from peripheral blood mononuclear cells (PBMC), obtained by Ficoll–Paque gradient density separation (GE Healthcare, Bio-Sciences AB, Uppsala, Sweden), as previously described in detail [34]. Monocyte preparations used in the experiments were >95% viable and >95% pure (assessed by trypan blue exclusion and cytosmears). Isolated monocytes included the subpopulations of classical, intermediate, and non-classical monocytes at the same percentages as present in PBMC, as indicated by the manufacturer and confirmed in-house by cytofluorimetric analysis of CD14- and CD16-expressing cells (Supplementary Figure S1). The staining procedure and flow cytometric analysis are reported in detail in the Supplementary Materials.

Monocytes were cultured in culture medium (RPMI 1640 + Glutamax-I; GIBCO by Life Technologies, Paisley, UK) supplemented with 50 µg/mL gentamicin sulfate (GIBCO) and 5% heat-inactivated human AB serum (Sigma-Aldrich, Inc., St. Louis, MO, USA). Cells (5–7.5 × 10^5^) were seeded in a final volume of 1.0 mL in wells of 24-well flat bottom plates (well internal diameter 15.6 mm; Corning^®^ Costar^®^; Corning Inc. Life Sciences, Oneonta, NY, USA) at 37 °C in moist air with 5% CO_2_. Monocyte stimulation was performed after overnight resting.

Freshly isolated monocytes were differentiated into tissue-like macrophages by culturing them in culture medium containing 50 ng/mL macrophage colony-stimulating factor (M-CSF; R&D Systems, Minneapolis, MN, USA) for 6 days (with one medium change on the third day). Differentiation and M2-like polarization, typical of tissue resident macrophages, was assessed morphologically and by the decreased expression of CD14 and increased expression of CD206. No significant mortality or increase in cell number was observed at the end of the differentiation period.

### 2.3. Human Cell Activation and Induction of Innate Memory

For assessing the primary response to stimulation, monocytes or macrophages were exposed for 24 h to LPS (positive control; from *E. coli* O55:B5 or *K. pneumoniae*; Sigma-Aldrich, Inc., St. Louis, MO, USA) or to increasing concentrations of rSmCD59.2, rSmTSP-2, unconjugated OMV, OMV:D, OMV:T, heat-killed *K. pneumoniae* (clinical isolates of both wild-type and carbapenemase-producing bacteria), or left untreated (medium/negative control).

For memory experiments, after the first exposure to stimuli for 24 h and supernatant collection, cells were washed and cultured with fresh culture medium for 6 additional days (one medium change after 3 days), to allow for the extinction of the activation induced by the previous stimulation. After this resting phase, the supernatant was collected, and cells were challenged for 24 h with fresh medium alone or containing a ten-fold higher concentration of stimuli. All supernatants (after the first stimulation, after the resting phase and after the challenge phase) were frozen at −20 °C for subsequent cytokine analysis. By visual inspection, cell viability and cell number did not substantially change in response to the different treatments.

### 2.4. Cytokine Analysis

The levels of the human inflammatory cytokines TNFα and IL-6 and of the anti-inflammatory factor IL-10 were assessed by ELISA (R&D Systems), using a Cytation 3 imaging multi-mode reader (BioTek, Winooski, VT, USA).

### 2.5. Statistical Analysis

Data were analyzed using the GraphPad Prism6.01 software (GraphPad Inc., La Jolla, CA, USA). For cytokine production, results are presented as ng produced cytokine/10^6^ plated monocytes. Results are reported as mean ± SD of values from 2 to 4 replicates from the same donor or from 2 to 4 different donors. Statistical significance of differences is indicated by *p* values, which were calculated using one-way non-parametric ANOVA with *post hoc* Tukey’s multiple comparison test and one-tailed unpaired *t* test.

## 3. Results

### 3.1. Particle Characterization

Unconjugated OMV and OMV–antigen complexes were characterized for their size, polydispersity, presence of recombinant antigens, and LPS content.

**Figure 1:**
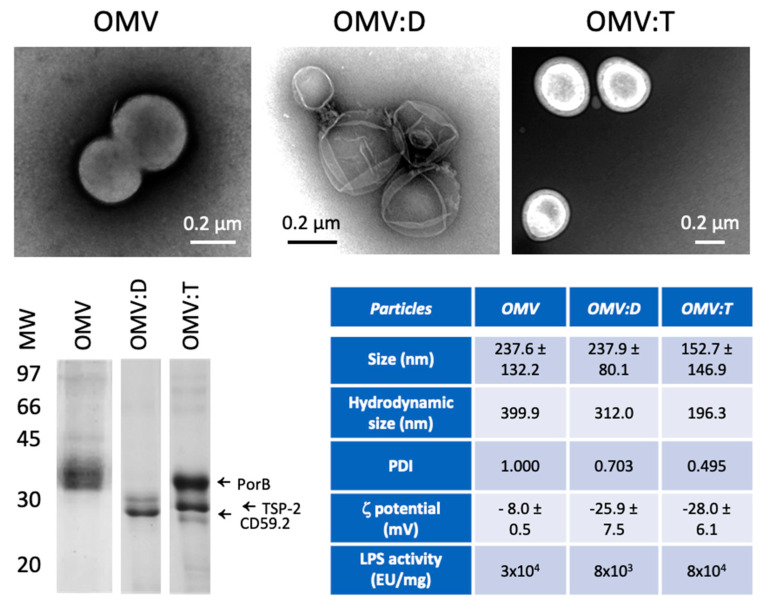
**Characterization of Outer Membrane Vesicles (OMV) and OMV–antigen complexes.** TEM images of unconjugated biotinylated OMV of *N. lactamica* (OMV, upper left), OMV conjugated with rRzvSmCD59.2 (OMV:D, upper center) and OMV conjugated with rRzvSmTSP-2 (OMV:T, upper right). Lower left panel: electrophoretic mobility of OMV and OMV–antigen complexes. Left arrows indicate the position of the main *N. lactamica* protein PorB, of SmTSP-2 in OMV:T and of SmCD59.2 in OMV:D (the two antigens having a calculated MW of 27.2 kDa for rRzvSmTSP-2 and 26.9 kDa for rRzvSmCD59.2). Lower right table: Summary of the OMV characteristics of size (measured in TEM, mean ± SD of 16–131 particles), hydrodynamic size, polydispersity, ζ-potential (mean of 3 determinations ± SD) (all measured by dynamic light scattering (DLS)) and LPS activity (measured with the LAL assay). PDI, polydispersity index.

The results in Figure 1 show that the three particles have similar characteristics, with an average size between 150 and 250 nm (corresponding to a hydrodynamic size of 200–400 nm) and a negative ζ-potential. All OMV preparations show polydispersity, which is likely due to both particle size heterogeneity and to aggregates [11], as confirmed by TEM. The presence of the recombinant antigens on the surface of OMV:D and OMV:T was confirmed to be about 1:10–1:20 vs. OMV proteins. A more complete characterization of the OMV, from isolating to the OMV–antigen conjugates, has been published elsewhere [10]. After detoxification, all OMV still displayed a residual LPS activity, which was at least 30 EU/μg in unconjugated OMV, 8 EU/μg in OMV:D, and 80 EU/μg in OMV:T. Since 1 EU roughly corresponds to 100 pg LPS, we can infer the presence of 3 ng LPS per μg particles in unconjugated OMV, 0.8 ng/μg in OMV:D, and 8 ng/μg in OMV:T (the latter value was confirmed by KDO assessment). It should be noted that the OMV complexes comply with the quality control criteria for OMV vaccines that require a residual LPS activity of <400 EU/μg.

### 3.2. Innate Response of Innate Immune Cells to S. mansoni Antigens

**Figure 2:**
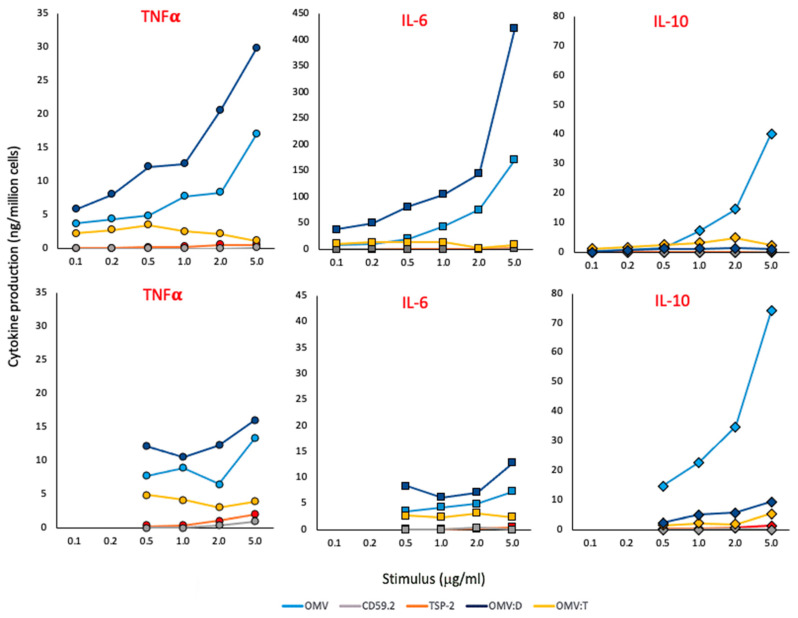
**Primary response of human monocytes and macrophages to *S. mansoni* antigens.** The production of TNFα (left panels, round symbols), IL-6 (center panels, square symbols) and IL-10 (right panels, diamond symbols) was assessed in human fresh blood monocytes (upper panels) and monocyte-derived macrophages (lower panels) stimulated for 24 h with increasing concentrations of unconjugated OMV (light blue), rSmCD59.2 (gray), rSmTSP-2 (red), OMV:D (dark blue), or OMV:T (yellow). The negative and positive controls (culture medium alone and LPS 10 ng/mL) are the following: TNFα in monocytes 0.03 and 2.23 ng/10^6^ cells; TNFα in macrophages 0.00 and 3.06; IL-6 in monocytes 0.04 and 5.00; IL-6 in macrophages 0.00 and 1.84; IL-10 in monocytes 0.00 and 0.16; IL-10 in macrophages 0.02 and 0.30. Data are from one donor of 2–4 tested (data from other donors are reported in the Supplementary Table S1). The SD of technical replicates was always <10% and is not reported. Statistical significance is reported in the Supplementary Table S2.

The capacity of recombinant *S. mansoni* antigens to stimulate the production of inflammatory (TNFα, IL-6) and anti-inflammatory (IL-10) cytokines was assessed on fresh human blood monocytes and on monocyte-derived macrophage (Figure 2). Both soluble antigens and antigens coupled to OMV were examined in parallel to unconjugated OMV and to the prototypical inflammatory stimulus LPS.

As shown in Figure 2, soluble antigens have little/no activity (gray and red symbols). Conversely, unconjugated OMV (light blue symbols) have a strong capacity of inducing inflammatory cytokines in monocytes and, to a lesser extent, in macrophages, while they are very potent inducers of IL-10 in monocytes and even more in macrophages. OMV:D (dark blue symbols) are more potent than unconjugated OMV in inducing inflammatory cytokines while essentially unable to induce IL-10. On the other hand, OMV:T (yellow symbols) have little/no activity, which is similar to the soluble recombinant protein. A possible interfering role for biotin, present on all OMV preparations for antigen ligation, is likely to be minimal/null, since the three OMV types showed variable qualitative and quantitative differences in the induction of different cytokines while containing the same amount of biotin.

The concentrations indicated in Figure 2 are those of the recombinant proteins, either alone or coupled to OMV; e.g., 1 μg OMV:T is the amount of OMV:T that contains 1 μg of SmTSP-2 (the amount of OMV being about 10× higher, i.e., 10 μg). Likewise, for 1 μg of unconjugated OMV, it is intended that the amount of OMV contained in complexes displaying 1 μg of Sm antigens (again 10 μg). Data in Figure 2 are from one donor, which are representative of two to four tested (the results from all donors are reported in the Supplementary Table S1).

### 3.3. Innate Memory of Human Monocyte/Macrophages to S. mansoni Antigens

**Figure 3:**
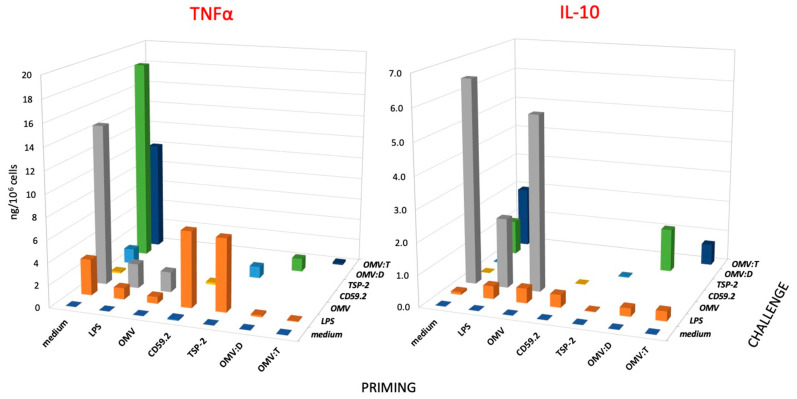
**Secondary response of human monocytes to challenge with *S. mansoni* antigens.** Production of TNFα (left panel) and IL-10 (right panel) of human monocytes that had been previously exposed (PRIMING in the horizontal axis) to culture medium alone (medium), LPS (1 ng/mL), unconjugated OMV (OMV), rSmCD59.2 (CD59.2), rSmTSP-2 (TSP-2), OMV:D, or OMV:T (all at 0.1 μg antigen/mL). After 6 days of resting, cells were challenged (see depth axis CHALLENGE) with a 10x higher concentration of stimuli; medium (purple), LPS (orange), OMV (gray), rSmCD59.2 (yellow), rSmTSP-2 (light blue), OMV:D (green) and OMV:T (dark blue). LPS was used as control challenge for cells primed with every kind of stimuli. Data are the values of the 24-h cytokine production by cells from one donor representative of three examined (see Supplementary Table S3 for the values of individual donors). SD of technical replicates were always <10% and are not shown. Statistical significance is reported in the Supplementary Table S4.

Monocytes were exposed for 24 h to a low concentration of Sm antigens, OMV, and OMV–antigen complexes (0.1 μg antigen/mL) or LPS as control (1 ng/mL) and cultured for an additional 6 days in fresh medium (one medium change after 3 days) to allow for return to a quiescent state. This was assessed by measuring the release of cytokines in the last 3-day supernatant, which was always undetectable (data not shown). At this time, monocytes had spontaneously differentiated in culture into macrophages. Cells were re-exposed to stimuli for 24 h, the challenge being a 10x higher concentration of LPS (10 ng/mL, as control) or the Sm antigens, OMV, and OMV–antigen complexes (1 μg antigen/mL). The inflammatory cytokine TNFα and the anti-inflammatory cytokine IL-10 were measured. The results in Figure 3 show the memory-induced variation in the cell response to different challenges, which are indicated with different colors. The data in Figure 3 refer to cells from a single donor out of three tested. Given the donor-to-donor quantitative variability of responses, the data could not be averaged (the results from each donor are reported in the Supplementary Table S2). In Figure 3, the response of unprimed cells is reported in the line indicated as “medium” in the horizontal axis “PRIMING”, and it shows that cells produce significant amounts of TNFα in response to LPS (orange), OMV (gray), and OMV–antigen complexes (green and dark blue), while the response to soluble rSmCD59.2 (yellow) and rSmTSP-2 (light blue) is limited. For IL-10 production, it is notable that unprimed cells do not respond well to LPS, while the response to OMV is very high. OMV–antigen complexes also induce IL-10 production, although to a lesser extent than bare OMV. This pattern of response reflects quite precisely the response of macrophages to a primary stimulation depicted in Figure 2.

When examining the memory response in terms of inflammatory TNFα production (Figure 3, left), we can observe that, as expected, priming with LPS induces a clear tolerance (decrease of response) to an LPS challenge, which is a phenomenon that can be observed also in cells primed with unconjugated OMV and OMV–antigen complexes, whereas priming with soluble Sm antigens slightly increased the secondary response to LPS (see orange columns, LPS challenge). Challenge with unconjugated OMV (gray columns) or with OMV–antigen complexes (green and dark blue columns) showed the same trend, i.e., a decreased TNFα production in cells primed with particulate agents as compared to unprimed cells. Conversely, the soluble Sm antigens triggered in primed cells the same low TNFα production as in unprimed control cells (yellow and blue columns). Thus, our data show that the particulate agents induce a tolerance-type memory in human monocytes/macrophages, which reduces the production of the inflammatory factor TNFα upon a secondary challenge (both identical and unrelated), while the soluble antigens do not have a significant effect. However, it should be noted that OMV also display a significant amount of LPS, roughly corresponding to the LPS control, which may imply that the tolerance effect of priming with OMV could be actually due to LPS.

Quite different is the picture of memory-induced modulation of the anti-inflammatory cytokine IL-10 (Figure 3, right). As already mentioned, challenge with LPS could induce a very limited production of IL-10 in unprimed cells, which was slightly increased in primed cells (except after rSmTSP-2 priming). In the case of priming with particulate agents (OMV, OMV:D, OMV:T), at variance with the results with TNFα, priming did not induce a clear tolerance-type response to challenge with either LPS or the identical agents, with only a partial decrease observed in the case of OMV:T homologous challenge. Thus, the memory response of particle-primed cells results in a significant decrease of the production of the inflammatory factor and no/little decrease of the anti-inflammatory factor, leading to a secondary response that is less inflammatory than that of unprimed cells. In the case of OMV:D, the response of unprimed cells to the complex (18.4 ng TNFα/10^6^ cells and 1.1 ng IL-10) was strongly rebalanced in OMV:D-primed cells (1.2 ng TNFα and 1.4 ng IL-10). In addition, in the case of OMV:T, the response of unprimed cells (10.0 ng TNFα and 1.9 ng IL-10, less inflammatory than the response to OMV:D) was significantly shifted toward anti-inflammation in primed cells (0.2 ng TNFα and 0.7 ng IL-10).

### 3.4. Innate and Memory Responses of Human Monocytes and Macrophages to Klebsiella pneumoniae LPS vs. Whole Bacteria

**Figure 4:**
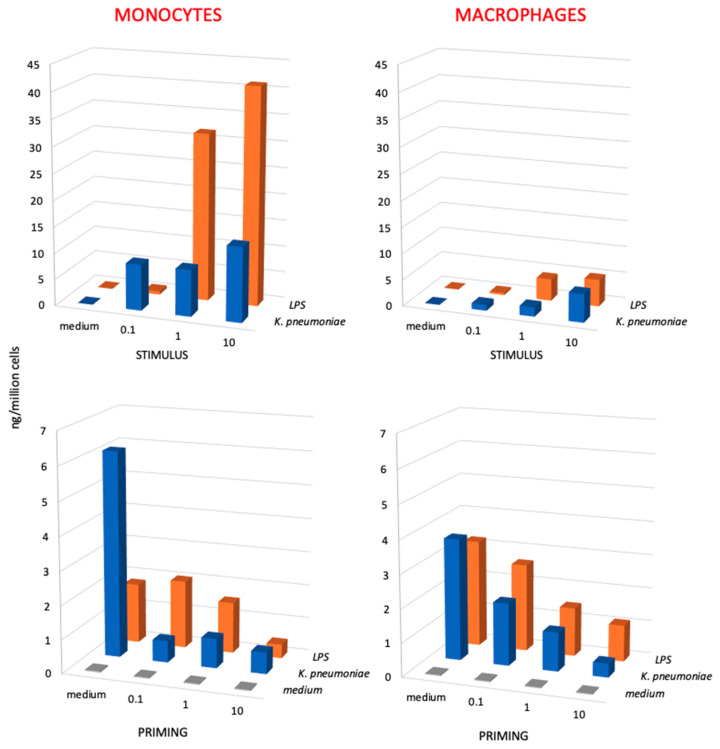
Primary and secondary innate response of monocytes and macrophages to *Klebsiella pneumoniae* bacteria vs. *K. pneumoniae* LPS. The primary response (upper panels) of monocytes (left panels) and monocyte-derived macrophages (right panels) was measured in terms of TNFα production upon stimulation with increasing concentrations of killed *K. pneumoniae* bacteria (0.1, 1, and 10 bacteria per each monocyte/macrophage; blue columns) and to LPS from *K. pneumoniae* (0.1, 1, and 10 ng/10^6^ monocytes/macrophages; orange columns). The secondary response (lower panels) of unprimed control cells (medium, in the horizontal axis; gray columns) and cells primed with increasing concentrations of *K. pneumoniae* bacteria (blue columns) or *K. pneumoniae* LPS (LPS; orange columns) was again measured in terms of TNFα production after a challenge of 24 h with the highest challenge concentration (10 bacteria per monocyte/macrophage; 10 ng LPS/10^6^ monocytes/macrophages). Only homologous priming/challenge combinations are shown (in the depth axis). Results are the average of two to four replicates from two different donors. SD were <20% and are not shown. Statistical analysis is reported in the Supplementary Table S5.

To assess the possible role of size on the capacity of microbial agents to stimulate innate responses and memory, we have tested the production of TNFα by human monocytes and monocyte-derived macrophages in response to LPS from Klebsiella pneumoniae in comparison to the whole inactivated bacteria, which display LPS on their surface. LPS concentrations were selected to correspond to those present on bacteria, considering that 1 EU (corresponding to about 100 pg LPS) is the amount of LPS displayed by 10^5^ bacteria. The results in Figure 4 (upper panels) show that the primary response of monocytes to LPS was 7–10× higher than that of macrophages, while the response of macrophages to K. pneumoniae was only half of that of monocytes at the highest concentration.

When examining the secondary memory response, both for LPS and for the whole bacteria, a tolerance-type memory response was evident, with the production of TNFα was much lower in primed vs. unprimed cells (Figure 4, lower panels). The secondary response of monocytes (lower left panel) represents the memory response of effector monocytes that entered an infected tissue and developed memory afterwards, whereas the secondary response of macrophages (lower right panel) represents the memory response of tissue-resident macrophages. The results show that the secondary response of monocytes, which respond to whole bacteria more potently than to isolated LPS, displays a potent tolerance when cells had been primed with whole bacteria, which is a tolerance that is already maximal at the lowest bacterial priming dose (ratio bacteria to monocytes 0.1 to 1). Conversely, tolerance to LPS depends on the LPS priming dose, being minimal at the lowest dose (0.1 ng LPS/10^6^ monocytes, corresponding to a bacteria to monocyte ratio of 0.1 to 1) and well evident only at a priming ratio of 10 to 1. A similar trend, although less pronounced, can be observed in the secondary response of tissue-like macrophages, in which the tolerance to challenge is significantly more pronounced in macrophages primed with increasing doses of bacteria in comparison to isolated LPS.

It should be said that in these experiments, we have used both wild-type *K. pneumoniae* and carbapenemase-producing *K. pneumoniae* isolates with identical results (the data in Figure 4 are the average values obtained in experiments with different isolates), which suggests that innate immunity and innate memory responses are not affected by the development of antibiotic resistance.

## 4. Discussion

The hypothesis that vaccination with a number of bacterial and viral vaccines, in particular those based on live attenuated microorganisms, may increase resistance to a number of unrelated diseases, in addition to the specific disease against which the vaccine is designed, is currently attracting significant attention for the possible positive impact on public health [24]. The biological basis for the non-specific effect of vaccination is most likely residing in the immunological phenomenon known as innate immune memory (or trained immunity), which is well known in plants and invertebrates and also present in vertebrates. Innate memory (also defined as “trained immunity”) implies a more efficient innate immune response to a challenge by organisms/cells previously exposed to the same or another agent [13,14,15,16,35,36,37]. In vaccine formulations, the specific immune response to antigens and the development of protective long-term adaptive immunological memory is facilitated by the use of adjuvants, which are agents that non-specifically induce a local inflammatory/innate immune reaction that creates the right conditions for the development of a potent and effective immunization against the vaccine antigens [38]. Indeed, epidemiological evidence supports the hypothesis that recurrent exposure to infectious stimuli is at the basis of a long-term protective immune memory that can be independent of T and B cells, thereby pointing at innate immune cells [39]. Thus, current research on vaccines aims at assessing the capacity of vaccine formulations (in particular their adjuvant components) not only to induce the immediate innate/inflammatory reaction required for optimal specific immunization but also to devise adjuvant strategies able to induce a non-specific innate memory that would increase the host resistance to a wider range of infections/diseases [22,23,24,25,26,40,41,42,43]. The concept of vaccination based on innate memory (“trained immunity” vaccines) is being further developed by the notion that organ/tissue-resident innate cells (in particular macrophages) can strongly contribute to innate memory-biased defensive responses to subsequent organ-specific infections [44,45,46,47,48].

In this study, we have investigated the possible role of innate memory, specifically focusing on mononuclear phagocytes (monocytes and macrophages), in the human reactivity to candidate vaccine formulations for *S. mansoni*. In fact, two promising parasite proteins (SmCD59.2 and SmTSP-2) are being developed in particulate formulations that provide excellent immunogenicity in experimental animals, with high production of antigen-specific antibodies and antigen-specific activation of CD4^+^ and CD8^+^ T lymphocytes [10,11]. The vaccine constructs imply the use of OMV from *N. lactamica*, which has been detoxified to decrease their endotoxin content below the acceptable limits for human use, and, using the MAPS technology, biotinylated and conjugated to recombinant fusion proteins encompassing the parasite protein in fusion with rhizavidin [10,11]. The particulate form, which is often used in vaccination strategies as effective antigen carrier, is known to be particularly effective in the interaction with macrophages, which react to particles by proliferating and by readily ingesting the particulate agents, thereby favoring their presentation to T lymphocytes for initiating adaptive immune responses (as macrophages are, together with dendritic cells, efficient antigen-presenting cells) [27,49,50]. Therefore, it is expected that particles, in addition to carrying the vaccine antigens, can have a direct capacity to initiate innate immune responses, at the basis of their adjuvant effect and, consequently, to induce the generation of an innate memory that can contribute to the vaccine efficacy.

The aim of this study was to examine the capacity of the two vaccine formulations for *S. mansoni* not only to activate innate immunity, i.e., to display an intrinsic adjuvant effect, but in particular if and how they can induce an innate immune memory able to contribute to long-term vaccine efficacy and non-specific resistance. To this end, we have examined the two *S. mansoni* antigens coupled with OMV (OMV:D and OMV:T) compared to unconjugated OMV and to the unconjugated SmCD59.2 and SmTSP-2 recombinant proteins. We should be aware of the fact that detoxified OMV still display endotoxin levels that, although below the threshold for regulatory approval, are detectable and active in our in vitro assays on human monocytes. Specifically, based on its activity the endotoxin levels may be of about 30 ng/mL for OMV, 8 ng/mL for OMV:D, and 80 ng/mL for OMV:T at the highest concentration used for challenge in memory experiments, i.e., 10 μg OMV (corresponding to 0.5–1.0 μg antigen)/mL, while 10x lower in the priming phase. Such endotoxin levels may be at least in part responsible for the capacity of OMV to induce an innate/inflammatory response and to establish a subsequent innate memory. The contribution of LPS in the innate/inflammatory activation of human monocytes and macrophages by OMV was assessed in comparison with similar amounts of purified LPS (from *E. coli*, since LPS from *N. lactamica* was not available; 1 ng/mL as priming and 10 ng/mL as challenge).

The results show different behaviors of OMV in inducing innate immune activation that cannot be exclusively attributed to LPS (see Figure 2). Indeed, OMV:T induced a primary response comparable to that induced by isolated LPS, but it was much lower than that triggered by unconjugated OMV and OMV:D. This is true for all inflammation-related factors examined, i.e., the inflammatory cytokines TNFα and IL-6 and the anti-inflammatory cytokine IL-10. Interestingly, OMV:D are more efficient than unconjugated OMV in inducing the two inflammatory factors, both in monocytes and macrophages, whereas the unconjugated particles are significantly more effective in inducing the anti-inflammatory factor IL-10, while LPS and all the other antigens show much lower effects. Thus, the innate reaction to OMV:T is low, in terms of induction of both inflammatory and anti-inflammatory factors, and at similar levels as the response induced by isolated LPS, while the soluble antigens do not induce any significant innate reaction. Interestingly, the unconjugated OMV induced a significant production of inflammatory factors but were also very effective in inducing the anti-inflammatory IL-10, suggesting that their innate immune activation potential and adjuvant effect may be limited. On the other hand, OMV:D are very efficient in inducing the inflammatory factors but not the anti-inflammatory cytokine, suggesting an efficient adjuvant effect. The inflammatory activation induced by the particles turns out to be transient, as expected for a safe adjuvant. Indeed, cell activation is completely extinguished after six additional days in culture, and cells have returned to a baseline quiescent state (again assessed in terms of cytokine production). Thus, we have shown that OMV induce a primary innate/inflammatory activation in both human monocytes and macrophages in culture that goes far beyond their content of LPS, in particular in quantitative terms, with only OMV:T showing effects quantitatively comparable to those of isolated LPS.

To stress the difference between the effect of LPS and that of LPS-bearing particles, we have also compared the innate activation induced in human monocytes and macrophages by different concentrations of LPS from *K. pneumoniae* in comparison to the entire *K. pneumoniae* bacteria displaying corresponding amounts of LPS on their surface (Figure 4, upper panels). In this case as well, bacteria have effects that are not superimposable to those of their LPS, as shown by the dose-independent activation of monocytes by whole bacteria compared to the strongly dose-dependent activation induced by isolated LPS. This causes a lack of response to a low dose of LPS, while the same dose of bacteria induces a significant response, and a very high response to intermediate and high LPS doses, while the response to the corresponding doses of bacteria is essentially identical to that triggered by the low dose and much lower than the response to LPS.

As already mentioned, the hypothesis that a priming of innate immunity could result in the establishment of a longer-term “innate memory” that contributes to an improved secondary reaction to a challenge (an infection, a second vaccine dose) has been recently explored by several groups and proposed as a very promising development in the vaccine field, in particular because of the non-specific effects of innate immunity that may afford protection against a wide spectrum of infections [22,23,24,25,26,40,41,42,43,44,45,47]. We have examined the possible role of innate memory upon challenge with the candidate *S. mansoni* vaccines by exploiting an in vitro system based on human primary monocytes that are exposed to the vaccine antigens twice in a priming–extinction–challenge sequence. Essentially, cells exposed in culture to the vaccine antigens for 24 h were subsequently allowed to rest for 6 additional days, so that their primary activation was extinguished and cells were again in a resting state. Then, cells were challenged with the same antigen (or LPS as control), and the response of antigen-primed cells was compared to that of cells that were not previously exposed (unprimed controls). In our study, we have examined the memory response in terms of production of two representative innate cytokines with opposing effects, the inflammatory TNFα and the anti-inflammatory IL-10, in order to have a realistic picture of the secondary innate reaction that includes the balance between inflammation and anti-inflammation. It should be noted that in this study, we have used a single low dose of each compound as priming stimulus, and a higher dose as challenge. This schedule derives from previous studies that have determined it to be the optimal way for assessing priming-induced innate memory, the concept being that memory induced by a lower-level primary stimulation (as in the case of vaccines) can induce a memory effect protective in the case of a strong challenge (e.g., an infection). To assess whether induction of innate memory is an hormesis phenomenon, i.e., whether low vs. high doses of a priming stimulus could afford opposite effects, we had examined the effect of different doses of priming stimuli on the ability to induce innate memory in vitro in human monocytes/macrophages, and we found that the memory effect was the same (tolerance) and dose-dependent, with increasing effect obtained with increasing priming doses, which was a finding that was true for a soluble stimulus, LPS, as well as for a particulate one, zymosan [50].

Examining the memory response in terms of TNFα production, we observed that priming with OMV (either unconjugated or coupled to antigens) results in a tolerance-like response to both a homologous challenge and to LPS. This means that the secondary response of primed cells is significantly lower than that of unprimed controls. This is the same kind of secondary response observed in LPS-primed cells, which reproduces the well-known phenomenon of LPS tolerance, aiming at preserving the host tissues from damage due to excessive inflammation upon repeated challenges [21,22]. Conversely, priming with the soluble antigens does not induce a different response to homologous challenge when compared to unprimed cells, whereas a potentiation of the response to LPS could be observed. The memory response in terms of production of the anti-inflammatory factor IL-10 shows a very different picture. Challenge with OMV (bare or antigen-decorated) can induce a potent production of IL-10 in control unprimed cells, which is an event that is not mimicked by LPS, showing that the OMV effect is most likely due to their particulate form rather than to their LPS content. Challenge with OMV also triggered a significant production of IL-10 in OMV-primed cells (homologous challenge), which was practically identical to that induced in unprimed cells (except for a partial decrease in OMV:D primed/challenged cells). Thus, while the particle-induced memory results in a tolerance effect for the inflammatory cytokine, the production of the anti-inflammatory cytokine is essentially not affected, suggesting an overall balance toward anti-inflammation in the secondary response. In fact, the TNFα/IL-10 ratios in unprimed vs. primed cells challenged with OMV:D are 16.7 vs. 0.9, and the TNFα/IL-10 ratios in unprimed vs. primed cells in response to OMV:T are 5.3 vs. 0.3, these figures being similar to those obtained with bare OMV (TNFα/IL-10 ratios in unprimed vs. primed cells in response to OMV are 2.2 vs. 0.3). Indeed, even in the case of LPS (a very good adjuvant apart from its toxicity), the TNFα/IL-10 ratio goes toward reduced inflammation in primed cells, being 33.0 (highly inflammatory) in unprimed cells challenged with LPS, and being significantly reduced to 2.5 in LPS-primed cells challenged with LPS. The priming effect of LPS is apparently changed by its presentation to monocytes/macrophages by whole bacteria, as shown in the experiments with *K. pneumoniae*. Monocyte priming with whole bacteria, even at the lowest dose, achieved a very profound tolerance effect (in terms TNFα production), whereas the tolerance induced by LPS was dose-dependent irrespective of the intensity of the primary response, suggesting that the LPS-bearing whole bacteria induce a more profound memory than the isolated agent.

The present study did not yet address the mechanisms underlying the observed memory effects (studies currently ongoing), which is an issue of particular interest since the observed effects were different (increased vs. decreased production) depending on the cytokine under evaluation, implying concomitant changes at different levels, likely including epigenetic and metabolic regulation [51,52,53,54,55].

## 5. Conclusions

These findings indicate that the OMV-based antigens can exploit the adjuvant effects of both the particulate form of OMV and the presence of non-toxic amounts of LPS, for shaping innate immunity and generating innate memory toward the amplification of immune responses with a safe, non-inflammatory long-term innate reactivity. Understanding the mechanisms at the basis of beneficial innate memory would allow us to modulate them in a controlled fashion in order to obtain the desired memory effects in future vaccination strategies as well as in therapeutic immunomodulation. Notably, the innate memory effects appear to be strongly dependent on the donor, which is most likely a consequence of their “immunobiography”, i.e., the cumulative effects of their individual history of exposure to repeated and different challenges during the lifetime [56]. This indicates the need for an individual immune memory profile as the basis for a personalized selection of the most appropriate (most effective and safer) preventive and therapeutic strategies.

## Data Availability

The data presented in this study are available in this article and its Supplementary Materials.

## Supplementary Materials

The following are available online at https://www.mdpi.com/article/10.3390/nano11040931/s1: Supplementary Materials and Methods (Staining Procedure, Flow Cytometric Analysis); Figure S1, Monocyte subsets before and after positive isolation with CD14 magnetic beads; Table S1, Primary cytokine production by human monocytes in response *to S. mansoni* antigens; Table S2, Statistical analysis of primary cytokine production by human monocytes in response to *S. mansoni* antigens; Table S3, Memory response of human monocytes from different donors primed with *S. mansoni* antigens; Table S4, Statistical analysis of memory responses of human monocytes primed with *S. mansoni* antigens; Table S5, Statistical analysis of primary and memory responses to *K. pneumoniae* bacteria and their LPS.
