# Supplementary material for: Primary and Memory Response of Human Monocytes to Vaccines: Role of Nanoparticulate Antigens in Inducing Innate Memory
Source: Nanomaterials (Basel). 2021 Apr 6;11(4):931. doi: 10.3390/nano11040931 (PMC8067467; doi:10.3390/nano11040931)
Supplement: Supplementary file 1 [file nanomaterials-11-00931-s001.pdf]

Supplementary Material

Primary and memory response of human monocytes to vaccines: role of nanoparticulate antigens in inducing innate memory

Mayra M. Ferrari Barbosa [1], Alex Issamu Kanno [1], Leonardo Paiva Farias [2], Mariusz Madej [3,†], Gergö Sipos [3], Silverio Sbrana [4], Luigina Romani [5], Diana Boraschi [3,6,*], Luciana C.C. Leite [1,*] and Paola Italiani [3,6,*]

[1] Laboratório de Desenvolvimento de Vacinas, Instituto Butantan, 05503-900 São Paulo, SP, Brazil; mayra_mara@msn.com (M.M.F.B.); alex.kanno@butantan.gov.br (A.I.K.)
[2] Laboratório de Inflamação e Biomarcadores, Instituto Gonçalo Moniz, Fundação Oswaldo Cruz, 40296-710 Salvador, Bahia, Brazil; leonardo.farias@fiocruz.br
[3] Istituto di Biochimica e Biologia Cellulare, Consiglio Nazionale delle Ricerche, 80131 Napoli, Italy; mariusz.madej@ocello.nl (M.M.); gergoosipos@hotmail.com (G.S.)
[4] Istituto di Fisiologia Clinica, Consiglio Nazionale delle Ricerche, 54100 Massa, Italy; silverio.sbrana@ifc.cnr.it
[5] Dipartimento di Medicina e Chirurgia, University of Perugia, 06132 Perugia, Italy; luigina.romani@unipg.it
[6] Stazione Zoologica Anton Dohrn, 80121 Napoli, Italy
* Correspondence: diana.boraschi@ibbc.cnr.it (D.B.); luciana.leite@butantan.gov.br (L.C.C.L.); paola.italiani@ibbc.cnr.it (P.I.)
† Current address: OcellO B.V., 2333 CH Leiden, The Netherlands

Supplementary Materials and Methods

Staining Procedure

The immunostaining was performed on fresh PBMC and monocytes, isolated as described in paragraph 2.2 from healthy donors. Cells were centrifuged at 300xg for 10 min, the supernatant fluid was discarded and the cell pellet was resuspended in cold PBS (Lonza Group Ltd. Basel, Switzerland) plus 1% BSA (Sigma-Aldrich, Inc., St. Louis, MO, USA). An adequate volume of cells was added to four different tubes ($1x10^6$ cells/tube) and incubated with the following antibodies in a final volume of 100 µl: tube 1, phycoerythrin (PE)-labelled isotype control (5 µl) + fluorescein isotiocyanate (FITC)-labelled isotype control (1 µl); tube 2, anti-CD14-FITC (10 µl) + isotype control-PE (5 µl); tube 3, anti-CD16-PE (10 µl) + isotype control-FITC (1 µl); tube 4, anti-CD14-FITC (10 µl) + anti-CD16-PE (10 µl). Isotype controls and specific antibodies were used at the same final concentration. All antibodies were from BD Biosciences (San Jose, CA, USA). Tubes were incubated for 30 min on ice in the dark, then diluted with 1 ml PBS/BSA 1% and centrifuged at 300xg for 10 min. Finally, samples were resuspended with 0.5 ml PBS/BSA 1% and immediately analysed by flow cytometry.

Flow Cytometric Analysis

Cells were analyzed for identification of the three subsets ($CD14^{++}CD16^{-}$ classical monocytes, $CD14^{++}CD16^{+}$ intermediate monocytes, $CD14^{+}CD16^{++}$ non-classical monocytes) by flow cytometry (FACScan, Becton Dickinson, Rutherford, NJ, USA) with BD Cell Quest software. Monocytes were initially gated using a morphological selection (gate) based on forward scatter (FSC, cellular size) and side scatter (SSC, cellular complexity) parameters. The subsequent evaluation of CD14 and CD16 expression was performed by quantitation of FL1 (green) and FL2 (orange) fluorescence emissions, which represent the specific binding of antibodies conjugated with the FITC and PE fluorochromes, respectively. Amplified settings for FSC and SSC were used in linear mode, and those for fluorescence channels were used in logarithmic mode. A threshold was fixed on FSC to exclude cellular debris. The analysis of CD14 and CD16 expression was performed both in PBMC and in purified monocyte suspension, with acquisition of 30,000 morphologically gated events per tube.

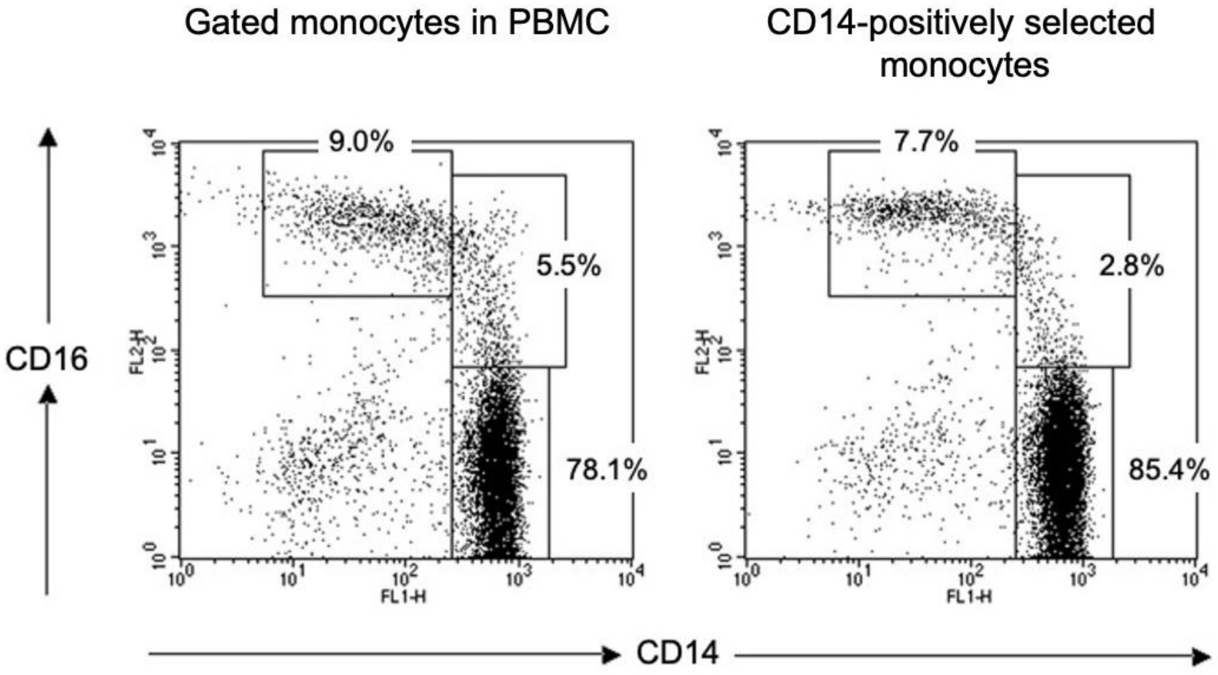

Figure S1. Monocyte subsets before and after positive isolation with CD14 magnetic beads. Scatter analysis of the surface markers CD14 and CD16 on monocyte subsets present in PBMC (left panel) and after magnetic purification with CD14 beads (right panel). Data are reported for the monocyte subset identification during the isolation procedure from blood of a representative donor. The percentage of classical $CD14^{++}CD16^{-}$, intermediate $CD14^{++}CD16^{+}$, and non-classical $CD14^{dim}CD16^{+}$ monocytes after magnetic purification fully reflected the percentage of the same monocyte subpopulations in PBMC. This indicates that the monocyte population used in our experiments is representative of the monocyte heterogeneity as present in the circulation.

Table S1. Primary cytokine production by human monocytes in response to *S. mansoni* antigens

Stimulus (µg/mL)		Cytokine production [1]																
		Monocytes										Macrophages						
		TNFα			IL-6				IL-10			TNFα		IL-6			IL-10	
		D1	D2	D3	D1	D2	D3	D4	D1	D2	D3	D1	D3	D1	D3	D4	D1	D3
medium		0.00	0.06	0.00	0.00	0.07	0.00	0.00	0.00	0.00	0.00	0.00	0.00	0.00	0.00	0.00	0.05	0.02
LPS	0.01	0.94	4.46	1.69	12.1	10.0	3.37	24.4	2.40	0.33	0.37	14.9	6.12	3.11	3.67	4.86	3.63	0.40
OMV	0.1	1.38	3.70	*nt*	12.1	7.88	*nt*	29.9	3.05	0.53	*nt*	16.2	*nt*	4.87	*nt*	4.28	5.38	*nt*
	0.2	1.14	4.34	*nt*	14.7	9.81	*nt*	29.5	4.95	0.56	*nt*	16.7	*nt*	3.71	*nt*	4.94	4.90	*nt*
	0.5	3.44	4.89	2.55	24.6	19.6	3.94	27.4	8.24	1.54	1.62	17.4	7.76	6.56	3.52	5.75	7.97	9.79
	1.0	5.73	7.77	2.33	46.5	43.3	7.61	47.9	11.7	7.18	17.4	17.9	8.94	9.19	4.32	4.25	10.7	15.1
	2.0	8.29	8.37	3.47	44.0	75.0	9.17	52.9	15.5	14.5	31.0	17.6	6.48	18.2	4.98	9.88	18.3	23.1
	5.0	*nt*	17.1	4.66	*nt*	170.6	1.59	101.3	*nt*	40.3	79.1	nt	13.4	nt	7.29	24.8	nt	49.4
SmCD59.2	0.1	0.00	0.00	*nt*	0.38	0.05	*nt*	0.00	0.00	0.00	*nt*	0.00	*nt*	0.00	*nt*	0.02	0.07	*nt*
	0.2	0.00	0.00	*nt*	0.00	0.35	*nt*	0.00	0.00	0.00	*nt*	0.14	*nt*	0.00	*nt*	0.03	0.10	*nt*
	0.5	0.01	0.00	*nt*	0.28	0.00	*nt*	0.03	0.00	0.00	*nt*	0.16	0.00	0.04	0.04	0.15	0.40	0.01
	1.0	0.02	0.00	0.17	0.14	0.00	0.01	0.04	0.00	0.00	0.06	1.79	0.00	0.19	0.02	0.19	0.57	0.00
	2.0	0.07	0.00	0.19	0.15	0.00	0.02	0.15	0.00	0.00	0.07	1.56	0.38	0.47	0.11	0.25	0.71	0.07
	5.0	*nt*	0.13	0.38	*nt*	0.19	0.06	*nt*	*nt*	0.00	0.00	*nt*	0.99	nt	0.13	0.43	nt	0.16
SmTSP-2	0.1	0.03	0.00	*nt*	0.10	0.11	*nt*	0.00	0.00	0.00	*nt*	0.02	nt	0.04	*nt*	0.12	0.19	*nt*
	0.2	0.04	0.00	*nt*	0.08	0.00	*nt*	0.02	0.00	0.00	*nt*	0.04	nt	0.11	*nt*	0.15	0.32	*nt*
	0.5	0.15	0.13	*nt*	0.33	0.10	*nt*	0.63	0.03	0.00	*nt*	0.95	0.37	0.39	0.09	0.32	0.62	0.19
	1.0	0.29	0.26	0.18	0.67	0.49	0.00	0.44	0.22	0.00	0.00	3.42	0.39	0.52	0.08	0.43	0.88	0.17
	2.0	0.40	0.52	0.21	0.78	0.66	0.07	0.98	0.46	0.00	0.19	9.29	1.08	0.70	0.19	0.52	1.01	0.43
	5.0	*nt*	0.51	0.68	*nt*	1.91	0.36	1.24	*nt*	0.11	0.42	*nt*	1.98	nt	0.54	0.66	nt	0.90
OMV:D	0.1	*nt*	5.80	*nt*	*nt*	37.1	*nt*	54.4	*nt*	1.17	*nt*	*nt*	*nt*	nt	*nt*	*nt*	6.99	*nt*
	0.2	*nt*	8.05	*nt*	*nt*	49.6	*nt*	37.0	*nt*	1.62	*nt*	*nt*	*nt*	nt	*nt*	*nt*	10.4	*nt*
	0.5	*nt*	12.2	*nt*	*nt*	80.5	*nt*	52.6	*nt*	2.44	*nt*	*nt*	12.1	*nt*	*nt*	8.35	7.41	1.53
	1.0	*nt*	12.6	5.27	*nt*	104.0	20.9	47.7	*nt*	3.08	2.61	*nt*	10.5	*nt*	*nt*	6.23	13.2	3.34
	2.0	*nt*	20.7	9.93	*nt*	144.4	37.6	45.5	*nt*	4.89	6.73	*nt*	12.3	*nt*	*nt*	7.18	16.3	3.82
	5.0	*nt*	29.8	12.0	*nt*	421.3	35.3	28.5	*nt*	2.36	5.75	*nt*	16.1	*nt*	*nt*	12.9	17.3	6.18
OMV:T	0.1	1.30	2.22	*nt*	23.0	10.3	*nt*	35.8	3.78	0.38	*nt*	7.72	*nt*	2.66	*nt*	5.71	4.35	*nt*
	0.2	1.69	2.74	*nt*	17.6	12.6	*nt*	41.2	3.71	0.58	*nt*	8.51	*nt*	4.78	*nt*	3.88	5.26	*nt*
	0.5	2.17	3.51	*nt*	29.5	13.0	*nt*	44.3	4.48	1.09	*nt*	9.24	4.84	4.57	2.67	6.80	5.64	1.02
	1.0	1.29	2.50	0.87	13.6	13.1	0.70	34.7	4.98	1.08	0.78	13.5	4.17	4.21	2.40	5.47	8.23	1.47

	2.0	0.18	2.16	0.89	9.87	1.75	0.83	20.6	4.89	1.26	1.57	10.6	3.05	4.73	3.15	4.27	5.98	1.19
0	5.0	*nt*	1.16	0.84	*nt*	8.25	0.07	4.69	*nt*	1.01	1.11	*nt*	3.89	*nt*	2.44	4.30	*nt*	3.53

[1] Cytokines were measured in the supernatant of monocyte and macrophage cultures from different donors (D1, D2, D3, D4) exposed for 24 h to culture medium alone (medium), LPS (10 ng/mL) or increasing concentrations of unconjugated OMV (OMV), the soluble *S. mansoni* antigens CD59.2 and TSP-2, and their complexes with OMV (OMV:D and OMV:T). Concentration of OMV, OMV:D and OMV:T are referring to the carried antigen rather than the overall OMV mass, which is about 10x higher. Results are the mean values of 2 technical replicates per each donor and are expressed in ng/10^6 cells. SD are always <5% and are not shown. Undetectable cytokine levels are shown as zero. *nt* = not tested.

Table S2. Statistical analysis of primary cytokine production by human monocytes in response to *S. mansoni* antigens

Sample 1	Sample 2	Monocytes			Macrophages		
		TNFα	IL-6	IL-10	TNFα	IL-6	IL-10
medium	LPS	0.001	0.05	ns	.0001	.0001	ns
	OMV 0.1	0.01	0.01	ns	*nt*	*nt*	*nt*
	OMV 0.2	0.001	0.01	ns	*nt*	*nt*	*nt*
	OMV 0.5	0.0001	0.05	ns	0.0001	0.0001	0.0001
	OMV 1.0	0.0001	0.01	0.0001	0.0001	0.0001	0.0001
	OMV 2.0	0.0001	0.05	0.0001	0.0001	0.0001	0.0001
	OMV 5.0	0.0001	ns	0.0001	0.0001	0.0001	0.0001
	CD59.2 0.1	ns	ns	ns	*nt*	*nt*	*nt*
	CD59.2 0.2	ns	ns	ns	*nt*	*nt*	*nt*
	CD59.2 0.5	ns	ns	ns	ns	ns	ns
	CD59.2 1.0	ns	ns	ns	ns	ns	ns
	CD59.2 2.0	ns	ns	ns	ns	ns	ns
	CD59.2 5.0	ns	ns	ns	ns	ns	ns
	TSP-2 0.1	ns	ns	ns	*nt*	*nt*	*nt*
	TSP-2 0.2	ns	ns	ns	*nt*	*nt*	*nt*
	TSP-2 0.5	ns	ns	ns	ns	ns	ns
	TSP-2 1.0	ns	ns	ns	ns	ns	ns
	TSP-2 2.0	ns	ns	ns	ns	ns	ns
	TSP-2 5.0	ns	ns	ns	0.05	ns	ns
	OMV:D 0.1	0.0001	ns	ns	*nt*	*nt*	*nt*
	OMV:D 0.2	0.0001	0.0001	ns	*nt*	*nt*	*nt*
	OMV:D 0.5	0.0001	0.0001	ns	0.0001	0.0001	ns
	OMV:D 1.0	0.0001	0.01	ns	0.0001	0.0001	0.0001
	OMV:D 2.0	0.0001	0.01	ns	0.0001	0.0001	0.0001
	OMV:D 5.0	0.0001	0.0001	ns	0.0001	0.0001	0.0001
	OMV:T 0.1	ns	ns	ns	*nt*	*nt*	*nt*
	OMV:T 0.2	ns	ns	ns	*nt*	*nt*	*nt*
	OMV:T 0.5	0.001	ns	ns	0.0001	0.0001	ns
	OMV:T 1.0	ns	ns	ns	0.0001	0.0001	0.05
	OMV:T 2.0	ns	ns	ns	0.0001	0.0001	ns
	OMV:T 5.0	ns	ns	ns	0.0001	0.0001	0.0001
OMV 0.1	OMV:D 0.1	ns	ns	ns	*nt*	*nt*	*nt*
	OMV:T 0.1	ns	ns	ns	*nt*	*nt*	*nt*
OMV 0.2	OMV:D 0.2	ns	ns	ns	*nt*	*nt*	*nt*
	OMV:T 0.2	ns	ns	ns	*nt*	*nt*	*nt*
OMV 0.5	OMV:D 0.5	0.0001	ns	ns	0.0001	0.0001	0.0001
	OMV:T 0.5	ns	ns	ns	.001	ns	0.0001
OMV 1.0	OMV:D 1.0	0.0001	ns	0.05	ns	0.01	0.0001
	OMV:T 1.0	0.0001	ns	0.0001	0.0001	0.01	0.0001
OMV 2.0	OMV:D 2.0	0.0001	ns	0.0001	0.0001	0.001	0.0001
	OMV:T 2.0	0.0001	ns	0.0001	0.0001	0.01	0.0001
OMV 5.0	OMV:D 5.0	0.0001	0.0001	0.0001	0.001	0.0001	0.0001
	OMV:T 5.0	0.0001	0.01	0.0001	0.0001	0.0001	0.0001
CD59.2 0.1	OMV:D 0.1	0.0001	ns	ns	*nt*	*nt*	*nt*
CD59.2 0.2	OMV:D 0.2	0.0001	ns	ns	*nt*	*nt*	*nt*
CD59.2 0.5	OMV:D 0.5	0.0001	0.05	0.01	0.0001	0.0001	0.05
CD59.2 1.0	OMV:D 1.0	0.0001	ns	ns	0.0001	0.0001	0.0001
CD59.2 2.0	OMV:D 2.0	0.0001	0.05	0.01	0.0001	0.0001	0.05
CD59.2 5.0	OMV:D 5.0	0.0001	0.0001	ns	0.0001	0.0001	0.0001
TSP-2 0.1	OMV:T 0.1	ns	ns	ns	*nt*	*nt*	*nt*
TSP-2 0.2	OMV:T 0.2	ns	ns	ns	*nt*	*nt*	*nt*
TSP-2 0.5	OMV:T 0.5	0.01	ns	ns	0.0001	0.0001	ns
TSP-2 1.0	OMV:T 1.0	ns	ns	ns	0.0001	0.0001	ns
TSP-2 2.0	OMV:T 2.0	ns	ns	ns	0.05	0.0001	ns
TSP-2 5.0	OMV:T 5.0	ns	ns	ns	0.05	0.01	0.0001

The table reports the *p* values (values are < of the indicated thresholds) obtained from the statistical analyisis of the experimental data in Figure 2 (2–4 replicates from one representative donor). The concentrations of the stimuli used, indicated in the first two columns, are in μg/mL (see Figure 2). ns = not significant; *nt* = not tested

Table S3. Memory response of human monocytes from different donors primed with *S. mansoni* antigens

Priming[1]	Challenge[2]	Cytokine production[3]					
		TNFα			IL-10		
		D1	D2	D3	D1	D2	D3
medium	medium	0.00	0.00	0.00	0.00	0.00	0.00
	LPS	4.20	2.43	3.25	0.19	0.20	0.08
	OMV	3.75	6.32	14.51	1.12	8.62	6.48
	SmCD59.2	0.08	2.04	0.15	0.00	0.22	0.00
	SmTSP-2	0.11	1.88	1.37	0.00	0.29	0.03
	OMV:D	9.49	10.20	18.43	1.39	1.18	1.11
	OMV:T	2.65	2.62	9.99	1.47	0.96	1.93
LPS	medium	0.00	0.00	0.00	0.00	0.00	0.00
	LPS	1.94	0.46	1.01	0.31	0.15	0.40
	OMV	*nt*	1.32	2.21	*nt*	1.32	2.21
OMV	medium	0.00	0.00	0.00	0.00	0.00	0.00
	LPS	1.29	0.51	0.00	0.23	0.20	0.46
	OMV	3.21	1.47	0.81	0.97	6.53	5.53
SmCD59.2	medium	0.00	0.32	0.00	0.00	0.05	0.00
	LPS	4.44	3.43	6.82	0.27	0.40	0.01
	SmCD59.2	0.00	1.96	0.20	0.00	0.19	0.00
SmTSP-2	medium	0.00	0.00	0.00	0.00	0.00	0.00
	LPS	3.72	2.39	6.54	0.00	0.18	0.02
	SmTSP-2	0.00	1.75	1.06	0.00	0.20	0.03
OMV:D	medium	0.00	0.00	0.00	0.09	0.00	0.00
	LPS	2.65	1.06	0.16	0.90	0.19	0.26
	OMV:D	5.72	2.99	1.22	0.65	0.81	1.38
OMV:T	medium	0.00	0.00	0.00	0.00	0.00	0.00
	LPS	0.72	0.52	0.06	0.29	0.26	0.30
	OMV:T	1.32	0.74	0.17	0.72	0.38	0.68

[1] Human monocytes were primed for 24 h with culture medium alone (medium), LPS (1 ng/mL), OMV (1.0 µg/mL), SmCD59.2 (0.1 µg/mL), SmTSP-2 (0.1 µg/mL), OMV:D (1.0 µg/mL, containing 0.1 µg antigen/mL) or OMV:T (1.0 µg/mL, containing 0.1 µg antigen/mL), then the stimuli were removed and fresh culture medium was added. [2] After priming, cells were cultured in the absence of stimuli for 6 days, then washed and challenged for 24 h with medium alone (negative control), LPS (10 ng/mL, positive control), OMV (10 µg/mL), SmCD59.2 (1.0 µg/mL), SmTSP-2 (1.0 µg/mL), OMV:D (10 µg/mL, containing 1.0 µg antigen/mL) or OMV:T (10 µg/mL, containing 1.0 µg antigen/mL). [3] Cytokine production was measured in the 24-h supernatant of challenged cells by specific ELISA and expressed as ng/10^6 cells. The values of three individual donors (D1, D2, D3) are reported, each being the average of two technical replicates. Undetectable cytokine levels are shown as zero. *nt* = not tested.

Table S4. Statistical analysis of memory responses of human monocytes primed with *S. mansoni* antigens

Sample 1 (priming/challenge)	Sample 2 (priming/challenge)	TNFα	IL-10
medium/medium	medium/LPS	0.0001	ns
	medium/OMV	0.0001	0.0001
	medium/CD59.2	ns	ns
	medium/TSP-2	ns	ns
	medium/OMV:D	0.0001	0.0001
	medium/OMV:T	0.0001	0.0001
LPS/medium	LPS/LPS	ns	0.01
	LPS/OMV	0.001	0.0001
OMV/medium	OMV/LPS	ns	0.01
	OMV/OMV	ns	0.0001
CD59.2/medium	CD59.2/LPS	0.0001	ns
	CD59.2/CD59.2	ns	ns
TSP-2/medium	TSP-2/LPS	0.0001	ns
	TSP-2/TSP-2	ns	ns
OMV:D/medium	OMV:D/LPS	ns	ns
	OMV:D/OMV:D	ns	0.0001
OMV:T/medium	OMV:T/LPS	ns	ns
	OMV:T/OMV:T	ns	0.0001
medium/LPS	LPS/LPS	0.001	ns
	OMV/LPS	0.0001	0.05
	CD59.2/LPS	0.0001	ns
	TSP-2/LPS	0.0001	ns
	OMV:D/LPS	0.0001	ns
	OMV:T/LPS	0.0001	ns
medium/OMV	LPS/OMV	0.0001	0.0001
	OMV/OMV	0.0001	0.0001
medium/CD59.2	CD59.2/CD59.2	ns	ns
medium/TSP-2	TSP-2/TSP-2	ns	0.05
medium/OMV:D	OMV:D/OMV:D	0.0001	ns
medium/OMV:T	OMV:T/OMV:T	0.0001	0.0001

The table reports the *p* values (values are < of the indicated thresholds) obtained from the statistical analysis of the experimental data in Figure 3 (2-4 replicates from one representative donor). ns = not significant.

Table S5. Statistical analysis of primary and memory responses to *K. pneumoniae* bacteria and their LPS

Sample 1	Sample 2	Monocytes	Macrophages
medium	*K.p.* 0.1	0.01	0.01
	K.p. 1.0	0.05	0.0001
	K.p. 10	0.0001	0.0001
	LPS 0.1	ns	ns
	LPS 1.0	0.0001	0.0001
	LPS 10	0.0001	0.0001
K.p. 0.1	*K.p.* 1.0	ns	0.01
	K.p. 10	ns	0.0001
	LPS 0.1	ns	0.01
K.p. 1.0	*K.p.* 10	ns	0.0001
	LPS 1.0	0.0001	0.0001
K.p. 10	LPS 10	0.0001	ns
LPS 0.1	LPS 1.0	0.0001	0.0001
	LPS 10	ns	.01
medium/medium	medium/*K.p.*	0.0001	0.0001
	K.p. 0.1/*K.p.*	ns	0.01
	K.p. 1.0/*K.p.*	ns	ns
	K.p. 10/*K.p.*	ns	ns
	medium/LPS	0.01	0.001
	LPS 0.1/LPS	0.01	0.001
	LPS 1.0/LPS	0.05	0.05
	LPS 10/LPS	ns	ns
medium/*K.p.*	*K.p.* 0.1/*K.p.*	0.0001	0.01
	K.p. 1.0/*K.p.*	0.0001	0.001
	K.p. 10/*K.p.*	0.0001	0.0001
	medium/LPS	0.0001	ns
K.p. 0.1/*K.p.*	*K.p.* 1.0/*K.p.*	ns	ns
	K.p. 10/*K.p.*	ns	.05
	LPS 0.1/LPS	ns	ns
K.p. 1.0/*K.p.*	*K.p.* 10/*K.p.*	ns	ns
	LPS 1.0/LPS	ns	ns
K.p. 10/*K.p.*	LPS 10/LPS	ns	ns
medium/LPS	LPS 0.1/LPS	ns	ns
	LPS 1.0/LPS	ns	0.05
	LPS 10/LPS	0.01	0.01
LPS 0.1/LPS	LPS 1.0/LPS	ns	ns
	LPS 10/LPS	0.01	0.05
LPS 1/LPS	LPS 10/LPS	0.05	ns

The table reports the *p* values obtained from the statistical analyisis of the experimental data in Figure 4 (2-4 replicates from two donors). The first part of the table refers to the primary response of monocytes and macrophages to different doses of *K. pneumoniae* bacteria (*K.p.*) and the corresponding doses of *K. pneumoniae* LPS (LPS) (upper panels of Figure 4). The second part of the table reports the comparisons of memory responses, in which each sample is defined by the combination of priming and challenge stimulations. Thus, as an example, medium/*K. p.* is the sample in which cells were primed with medium and challenged with *K. pneumoniae* bacteria. The dose of *K. pneumoniae* bacteria and LPS at challenges are fixed (10; *i.e.*, 10 bacteria per cell in the case of *K. pneumoniae* bacteria, and 10 ng/mL in the case of LPS) and are not indicated in the table, while the different doses used at priming are shown. ns = not significant.
